# Simultaneous Pulmonary Embolism and Carotid Thrombosis as a Presenting Manifestation of COVID-19

**DOI:** 10.1155/2022/4933172

**Published:** 2022-08-23

**Authors:** Leon Smith, Brian Zeman

**Affiliations:** Department of Rehabilitation Medicine, Royal North Shore Hospital, St Leonard's, NSW, Australia

## Abstract

Although COVID-19 was initially described predominantly as a respiratory infection, subsequent reports noted that it can produce generalised inflammation with effects on multiple organ systems. As a result, it is possible for patients with COVID-19, including those with severe disease, to present initially with nonrespiratory signs and symptoms. Neurological manifestations, including ischaemic stroke, may be the first presenting issue and can result from carotid thrombosis. Similarly, the risk of both arterial and venous thrombosis is increased in COVID-19, which may result from hypercoagulability associated with systemic inflammation.

## 1. Introduction

We describe a case of simultaneous pulmonary embolism and carotid thrombosis leading to multiterritory ischaemic stroke in an otherwise healthy, 62-year-old Indigenous Australian man.

## 2. Case Presentation

A 62-year-old man of Indigenous Australian background presented to hospital with reduced level of consciousness, dense right hemiplegia, and expressive aphasia. He lived in shared accommodation and was last seen well 3 days previously. The history was that at a welfare check, he was found semiconscious. At triage, his GCS was 11. He had respiratory rate of 40 breaths per minute, temperature of 38.1 C, oxygen saturation was 96%, and heart rate was 114 beats per minute. He was alert but aphasic and had right hemiparesis involving the arm and leg, right hypertonia, and hyperreflexia with positive right Babinski reflex. Due to his aphasia, history was limited and he had no available contacts or regular medical practitioner to obtain other information. Health department records included consultations four years previously for right leg cellulitis. It was reported at the time that he smoked tobacco and occasional marijuana and methamphetamine. Subsequent review of records from another hospital indicated a history of DVT three years previously. He had recently received his first dose of the AstraZeneca COVID-19 vaccine five days prior to admission.

### 2.1. Investigations

State health policy at the time was to perform COVID testing on patients who were febrile or who had respiratory symptoms. The patient was tested for COVID-19 with the Xpert Xpress SARS-CoV-2 real-time PCR assay. This test was positive, and the patient was isolated with airborne precautions thereafter. Initial blood tests revealed a white cell count of 17 × 10^9^/L, CRP of 80 mg/L, platelet count of 377 × 10^9^/L, and creatinine of 121 *µ*mol/L.

**Figure 1:**
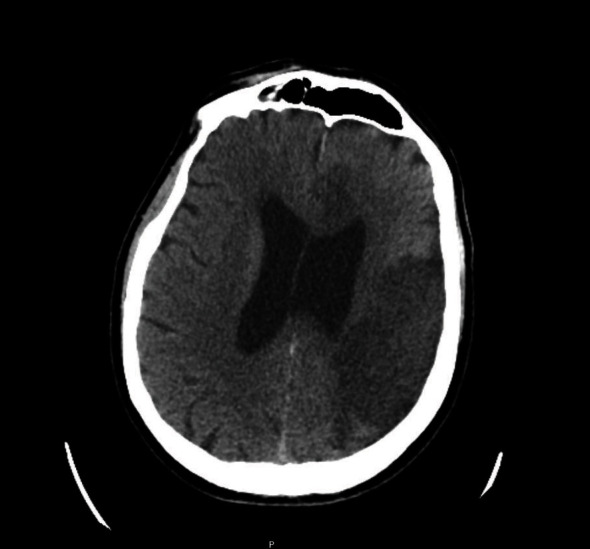
Noncontrast CT brain performed at the time of initial presentation, revealing established infarcts in the left MCA and ACA territories.

CT brain and CT angiography-circle of Willis scanning revealed extensive low attenuation in the left MCA and ACA territories, suggestive of extensive ischaemic infarction in these areas (Figure 1). Contrast angiography revealed diminutive M2 branches of the left MCA which appeared intact. No CT evidence of occlusive thrombus was present. The stroke team was involved, and as part of the acute stroke pathway, he was transferred to a tertiary referral hospital. He was admitted to their high dependency unit (HDU) for treatment of the multiterritorial ischaemic infarcts including intracranial pressure monitoring and treatment of COVID-19.

**Figure 2:**
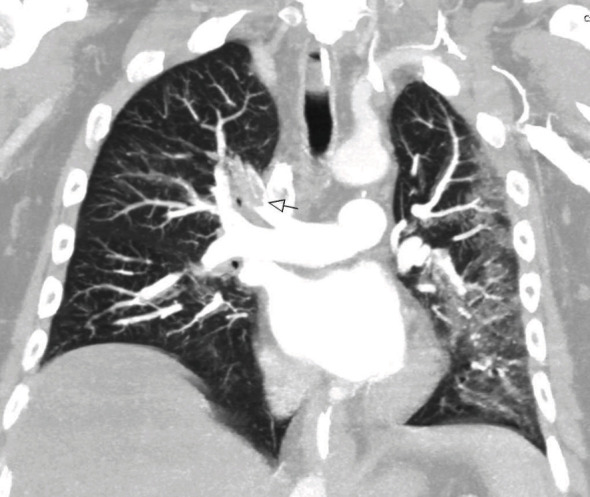
CT pulmonary angiogram performed on day 3 showing right-sided proximal segmental pulmonary embolism.

Initial chest X-ray showed possible “increased density” on the left lung. Chest X-ray on day 3 confirmed left-sided consolidation. Around this time, he required oxygen supplementation to maintain saturations above 95%. A CTPA was performed to “rule out” pulmonary embolism in view of recent immobility for 3 days and active COVID-19. The CTPA revealed extensive bilateral segmental and subsegmental pulmonary emboli, along with left lower lobe collapse and consolidation, with bilateral patchy ground-glass change (Figure 2).

**Figure 3:**
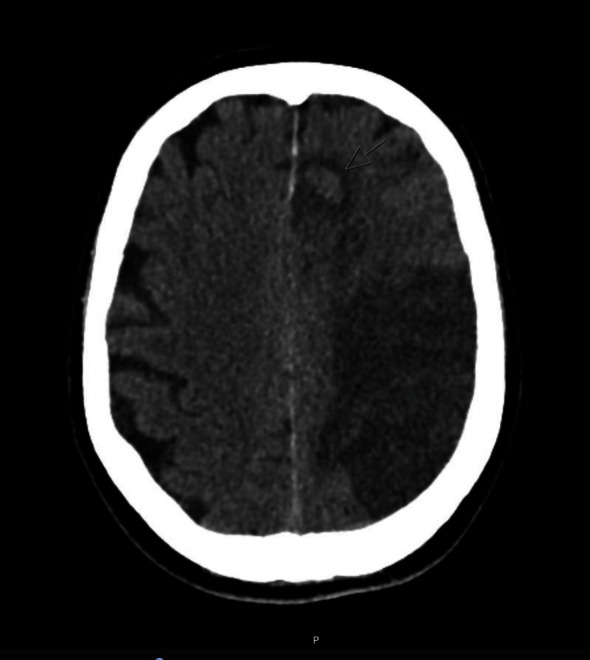
Noncontrast CT brain performed on day 3, revealing haemorrhage in the ACA territory.

Progress CT brain on day 3 showed petechial haemorrhage in the left frontoparietal MCA territory consistent with haemorrhagic transformation of affected area, with slightly increased mass effect and subfalcine herniation (Figure 3). A decompression hemicraniectomy was considered, although a decision was made for conservative management as the patient's clinical condition suggested against life-threatening raised intracranial pressure. Carotid duplex scanning performed on the same day showed left carotid sinus thrombus causing significant diameter reduction, with a mobile appearance. This was not present on the initial CT angiogram which did not extend proximally enough to cover the carotid sinus. It was initially felt that this could represent a paradoxical embolism, although cardiac abnormalities were subsequently excluded with echocardiography.

Standard investigations for the cause of the stroke were unremarkable, with normal random BSL, normal HBa1c, and normal fasting lipids. He was normotensive. Transthoracic echocardiography was essentially normal with normal valvular function, no shunt, and no other abnormal findings. Transoesophageal echocardiography was declined due to the aerosol-generating nature of the study. 24-hour Holter monitoring showed sinus rhythm. Other investigations for a secondary cause of the stroke or pulmonary emboli included tuberculosis gamma interferon release assay and serology for HIV and hepatitis B, all of which were negative. Antinuclear antibody was detected with a weakly positive titre of 1 : 40. Coagulation studies taken before anticoagulation was commenced were within normal limits. Thrombophilia screening was performed including for lupus anticoagulant, antithrombin III levels, protein S, functional protein C, and screening for factor V Leiden and prothrombin variant G20210A. These investigations all yielded normal results.

As his platelet counts were consistently within normal limits, he did not meet criteria for testing for Vaccine Induced Thrombosis with Thrombocytopenia Syndrome (VITTS). In accordance with guidelines for use of VITTS testing in use at the time, further evaluation for this condition was not performed.

### 2.2. Treatment

The patient was treated for COVID-19 in accordance with local protocol with dexamethasone for 7 days. The treating neurology team liaised with the COVID-19 team regarding the use of baricitinib; this was commenced on day 5, but continued only for 3 days before being ceased due to a possible increase in thromboembolism associated with baricitinib. Ceftriaxone and doxycycline were also used to treat a likely superimposed bacterial infection in view of the consolidation on chest X-ray.

In view of the large cerebral infarct volume with associated haemorrhagic transformation, anticoagulation was not given for the pulmonary emboli or carotid thrombus. Instead, an inferior vena cava (IVC) filter was inserted on day 3 of admission. Aspirin and prophylactic enoxaparin (40 mg at night) were given. Anticoagulation for the carotid thrombus was initially considered unsafe in view of the haemorrhagic transformation. MRI brain was contraindicated due to the presence of the IVC filter. When the haemorrhagic transformation had resolved (confirmed on CT brain on day 38 of admission), anticoagulation with apixaban was commenced along with aspirin. Atorvastatin was added for stroke secondary prevention. After 7 days, a repeat SARS-CoV-2 PCR test was negative and the patient was deisolated after two more PCR tests 24 hours apart.

### 2.3. Outcome and Follow-Up

Once stabilised, the patient was transferred to a general medical ward. He was initially poorly responsive and fully dependent. He improved gradually and was able to take oral food so that his nasogastric tube could be removed. He was referred for rehabilitation. At that stage, he had some active movement in the right side and was able to follow one-step commands but had minimal verbal output. The IVC filter was removed, and he was then admitted to the rehabilitation unit for ongoing multidisciplinary care including functional assessment, mobility retraining, and discharge planning. It was recommended that he remain on lifelong anticoagulation.

He was admitted to rehabilitation on day 70. Initial FIM score on admission to rehabilitation was 24. There was limited improvement after 3 weeks, and he was put onto a maintenance program whilst awaiting discharge to supported accommodation. At the time of discharge, he had ongoing right hemiplegia, with no functional use of right arm or leg. He required a hoist transfer, being unable to stand independently. He could sit in a wheelchair, but his interest in self-mobilisation was limited. In terms of cognition and communication, he was dysphasic with limited verbal output, although was able to follow at least two-step commands. He could indicate his understanding of simple treatments or information and was judged as being able to give consent or refuse procedures or investigations, although for more complex decisions, the support of a guardian was required. He could feed himself, but required assistance to dress and shower and required assistance with continence management.

A repeat carotid arterial doppler performed on day 76 showed resolution of the carotid thrombus, with minimal carotid stenosis. An MRI was performed on day 80 after the IVC filter had been removed; this confirmed the presence of the previously described infarcts. The patient had received quadrivalent influenza immunisation 3 months before this admission, as well as his first dose of the AstraZeneca COVID-19 vaccine. A second dose was given three months later, prior to transfer to rehabilitation, with no adverse effects.

## 3. Discussion

Assessing the causal relationship between this patient's infection and subsequent thrombotic events is not straightforward, as the specific onset date of infection is not well defined. However, he tested negative for SARS-CoV-2 by PCR by day 7. The average duration of PCR positivity is believed to be at least 10 days, suggesting that our patient would have been infected for some days prior to his presentation [1, 2]. This suggests that his infection preceded the stroke. This does not establish a causal relationship definitively, although the occurrence of similar presentations (including with multiterritorial infarcts) suggests that such a relationship is at any rate possible [3].

Patients hospitalized with COVID-19 have a high prevalence of pulmonary embolism [4], with up to 14% of those admitted to hospital with COVID-19 having pulmonary embolism on systematic screening with CTPA [5] or 25% of those clinically suspected of having PE being confirmed to have it [6]. The prevalence of carotid thrombosis in COVID-19 has been reported in the literature [7–10] and appears that COVID-19 is an important risk factor for ischaemic stroke [11]. It is increasingly recognized that COVID-19 is associated with a hypercoagulable state [12–14] which can manifest as stroke and DVT/PE [15]. Several possible mechanisms for this have been proposed, including local pulmonary inflammation leading to a subsequent coagulation cascade with systemic thrombosis elsewhere [13]. Endothelial damage has also been reported as a consequence of COVID-19 [16]; the combination of endothelial injury and hypercoagulability itself increases the risk of venous thrombosis. In conjunction with reduced mobility (as was the case in our patient), this completes Virchow's triad of risk factors for venous thromboembolism [17]. The pathogenesis of arterial thrombosis in COVID-19 is less well understood, although the inflammatory state associated with COVID-19 is likely to be involved, particularly the “cytokine storm” associated with systemic infection [9].

The role of the AstraZeneca vaccine in this case requires comment, as the patient's presentation occurred within days of being administered the vaccine. He did not meet criteria for VITTS-specific testing in use at the time and so did not undergo platelet-factor 4 testing or intravenous immunoglobulin treatment. Subsequent revisions to the guidelines introduced since his admission would have considered this case as “less likely” to be VITTS and may have justified nonurgent VITTS testing. In either case, his normal platelet counts and a lack of adverse response to enoxaparin suggest against VITTS being a factor in this case.

Our patient had a documented history of cellulitis some years prior to this presentation in 2017 and a later presentation in 2018 with DVT. He was a smoker. No specific condition increasing his risk for a subsequent DVT was apparent from his available medical history, although the occurrence of a previous DVT is in itself a significant risk factor for repeat thrombosis. Reduced mobility from stroke could have increased the risk of a venous thrombosis, particularly in the presence of clinical and biochemical evidence of a “long lie,” although this would not itself explain the pathogenesis of the arterial thrombosis, which likely precipitated stroke.

This case had several challenges due to high level of physical dependency, social and accommodation issues, aboriginal background (an ethnic group at higher risk of unfavourable COVID-19 outcomes) [18], lack of family (subsequently requiring a public guardian for complex decisions), and delays due to need to involve the Australian National Disability Insurance Scheme. His motivation to participate fluctuated throughout admission. There was no apparent evidence of depression, but he was assessed by a clinical psychologist to confirm that he was not depressed.

This case is of someone who despite being under 65 was at risk from developing COVID and getting complications. He was of aboriginal and low socioeconomic background, living in shared accommodation, and was a smoker. He had the history of substance abuse and DVTs.

## 4. Conclusion

We report on a case of carotid thrombus leading to multiterritorial ischaemic stroke, along with bilateral pulmonary embolism in a patient with COVID-19 and no other significant risk factors for carotid thrombosis. The COVID-19 pandemic is likely to increase the risk of unusual thrombotic events, including simultaneous arterial and venous thrombosis, in patients who otherwise have no specific risk factors for the same. A low threshold of suspecting COVID-19 in patients with unusual thrombotic events is advised.

The focus on issues related to microbiology, haematology, and neurology is of importance, particularly initially. However, associated social and long term issues need to be considered in complex COVID-19 patients including the impact of the pandemic on individuals who are at risk and their needs to return to the community.

## 5. Disclosure

This study was performed as part of the authors' employment at Northern Sydney Local Health District, NSW Australia. The employer was not involved in manuscript writing, editing, and approval or decision to publish.

## Data Availability

Patient information was accessed through medical records at Royal North Shore and Ryde Hospitals and is unavailable for release due to patient confidentiality.
